# Identification and characterization of miRNAs in spleens of sheep subjected to repetitive vaccination

**DOI:** 10.1038/s41598-023-32603-7

**Published:** 2023-04-17

**Authors:** Endika Varela-Martínez, Martin Bilbao-Arribas, Naiara Abendaño, Javier Asín, Marta Pérez, Lluís Luján, Begoña M. Jugo

**Affiliations:** 1 Department of Genetics, Physical Anthropology and Animal Physiology, Faculty of Science and Technology, University of the Basque Country (UPV/EHU), Sarriena auzoa, 48940 Leioa, Spain; 2 Department of Animal Pathology, Veterinary Faculty, University of Zaragoza, Zaragoza, Spain

**Keywords:** Computational biology and bioinformatics, Immunology, Systems biology

## Abstract

Accumulative evidence has shown that short non-coding RNAs such as miRNAs can regulate the innate and adaptive immune responses. Aluminium hydroxide is a commonly used adjuvant in human and veterinary vaccines. Despite its extended use, its mechanism of action is not fully understood and very few in vivo studies have been done to enhance understanding at the molecular level. In this work, we took advantage of a previous long-term experiment in which lambs were exposed to three different treatments by parallel subcutaneous inoculations with aluminium-containing commercial vaccines, an equivalent dose of aluminium or mock injections. Spleen samples were used for miRNA-seq. A total of 46 and 16 miRNAs were found differentially expressed when animals inoculated with commercial vaccines or the adjuvant alone were compared with control animals, respectively. Some miRNAs previously related to macrophage polarization were found dysregulated exclusively by the commercial vaccine treatment but not in the aluminium inoculated animals. The dysregulated miRNAs in vaccine group let-7b-5p, miR-29a-3p, miR-27a and miR-101-3p are candidates for further research, since they may play key roles in the immune response induced by aluminium adjuvants added to vaccines. Finally, protein–protein interaction network analysis points towards leucocyte transendothelial migration as a specific mechanism in animals receiving adjuvant only.

## Introduction

microRNAs (miRNAs) are ~ 21 nucleotide long non-coding RNAs that play key roles in gene regulation through translational repression or mRNA decay. miRNAs usually bind by sequence complementarity of their seed sequence (nucleotides 2–7) to the 3’ UTR sequence region of a target gene^1^. Accumulative evidence has shown that miRNAs can regulate the innate and adaptive immune responses. For instance, miR-155 has been shown to be essential for regulation of T helper cell differentiation^2^, miR-142 has been shown to be pivotal for metabolic reprogramming of dendritic cells (DCs)^3^ and the miR-125 family has been related to macrophage polarization^4^. Accordingly, miRNAs may have essential roles in the immune response to vaccines and adjuvants. Studies have shown that miRNAs affect immune responses after vaccination and vaccine efficacy in both, innate response blood cells and adaptive response cells from lymphoid organs^5^. In humans, several miRNAs were shown to affect the response to an inactivated virus vaccine^6^ and some miRNAs are correlated with antibody titers after vaccination with a mRNA vaccine for COVID-19^7^.

Most of the miRNA-related research has been performed with vaccine formulations based on pathogen components as endogenous adjuvants, but many other vaccines use sterile substances as adjuvants, which activate immune pathways differently^8^. Adjuvants can be described as compounds added to vaccines to enhance or modulate the immune response. Aluminium-based adjuvants, and specifically aluminium hydroxide, are one of the most used adjuvants in human and animal commercial vaccines due to their excellent safety profile, minimal reactogenicity and inexpensiveness^9^. Despite their extended use, the mechanism of action by which they elicit an immune reaction is not fully understood^10^.

After vaccination with aluminium adjuvants, aluminium particles are phagocytosed by cells of monocytic lineage and are transported to draining lymph nodes (DLN), bloodstream and other secondary lymphoid organs^11^. In the lymph nodes (LNs), those cells can transfer antigen material to a network of antigen presenting cells (APCs) and induce the activation of the immune system in distant organs such as spleen^12^. The spleen is the largest secondary lymphoid organ and apart from its role in blood filtration, it functions similarly to a LN by regulating T and B cell responses to antigenic targets in the blood^13^. Thus, vaccine components reach the spleen through the bloodstream or transported by APCs.

We previously profiled the gene and miRNA expression in sheep PBMCs^14^ and encephalon^15^ after an experiment in which animals were repetitively inoculated with commercial vaccines or aluminium hydroxide alone. In this work, we sequenced the spleen miRNA transcriptome of animals from the same experiment in order to characterize the miRNAome in an ovine secondary lymphoid organ and assess the role miRNAs may play in the immune response of commercial vaccines and aluminium adjuvants.

## Results

### Summary statistics

Twelve spleen samples were sequenced, four from each group (vaccine, adjuvant and control). A detailed summary per sample of the alignment and miRNA characterization can be seen in Table S1. Briefly, a mean sequencing depth of 14.9 (± 2.2) million reads was achieved. After adapter trimming, quality filtering and rRNA removal a mean of 13.3 (± 2.0) million reads remained for further analysis. Surviving reads were aligned to the *Ovis aries* reference genome (Oar_rambouillet_v1.0), of which approximately 80% aligned.

### Expression profile of spleen miRNAs

**Figure 1:**
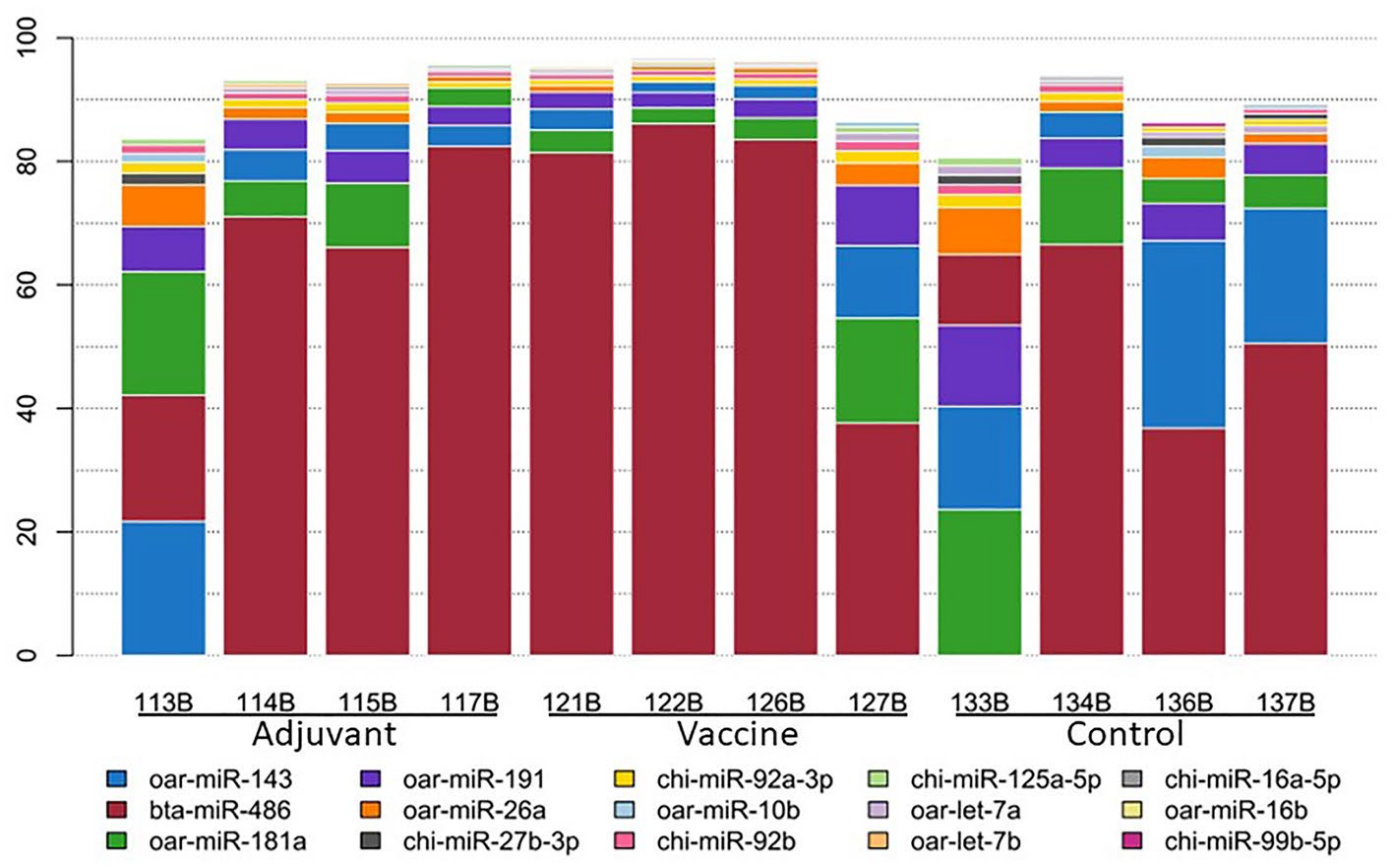
The 10 most highly expressed miRNAs in each sample. In the x-axis the samples and in the y-axis the percentage of the total reads for each miRNA. The bars are coloured by miRNAs.

After alignment to the reference genome, srnatoolbox was used for miRNA characterization. First, miRNAs were characterized using the ovine, caprine and bovine miRNAs from miRBase, with the priority of search ovine > caprine > bovine. A total of 394 miRNAs (95 ovine, 200 caprine, 55 bovine and 44 novel) were expressed with at least one sequence read count in at least one of the samples. Of the 22 novel pre-miRNAs, 18 had a similar seed sequence to annotated miRNAs. Those miRNAs with an expression of 1 cpm in at least four samples were taken as expressed and 278 miRNAs remained for further analysis. Interestingly, 8 out of 12 samples had a higher proportion of reads aligning to bovine miRNAs (Supplementary Table S1). After the expression counts were obtained for each miRNA, the 10 most highly expressed miRNAs were found to take approximately 80 to 90 percent of the aligned counts (Fig. 1). A handful of miRNAs were also highly expressed in the other tissues previously studied from the same experimental group. The 10 most expressed miRNAs in PBMCs^14^ and parietal lobe cortex data^15^ constitute most of the aligned reads (Supplementary Figs. S1 and S2), but in the spleen this distribution was even more extreme. A single miRNA, bta-miR-486, was highly expressed in most animals, taking up to 80 percent of the aligned reads in some samples. This miRNA expression profile would explain why most samples had a higher proportion of reads aligning to bovine miRNAs.

The miRNAs detected in spleen in this study were compared with those detected in PBMCs and parietal lobe cortex (Supplementary Fig. S3). The PBMC samples were reanalysed due to differences in the pipeline and the same protocol as the one described in this work was applied. Interestingly, 110 miRNAs were detected above the expression threshold in all tissues. The spleen data showed the highest number of exclusive miRNAs with 140 (50%), followed by the encephalon data with 104 miRNAs (40%), and only 19 miRNAs (14%) were exclusive to PBMCs.

### Differential gene expression analysis

Prior to the differential expression analysis, a principal component analysis (PCA) was performed (Supplementary Fig. S4). DESeq2 was used for the differential expression analysis and a total of 46 miRNAs were found differentially expressed between the Vaccine and Control sheep, including 19 upregulated miRNAs and 27 downregulated miRNAs such as miR-29a, miR-27a and miR-101-3p (Table 1). Furthermore, 16 miRNAs were found differentially expressed between the Adjuvant and Control groups, among them 6 upregulated miRNAs and 10 downregulated such as miR-451-5p (Table 2). Twelve miRNAs were concordant with the same log2 fold change sense in both comparisons. The most significantly differentially expressed miRNA in both comparisons was miR-148b-3p, which belongs to the miR-148/-152 family. At last, four miRNAs were downregulated by both treatments: miR-30c, miR-126-5p, miR-30e-3p and miR-186-5p. Only four differentially expressed miRNAs were exclusive of the adjuvant-only treatment. . We did not find any DE miRNA between both treatment groups.

**Table 1:** Differentially expressed miRNAs in the Vac vs. Control comparison. baseMean: average of the normalized DESeq2 count values.

Type	miRNA	baseMean	log2FoldChange	padj
Up-regulated	oar-miR-485-5p	54.47	1.46	1.279E − 02
	bta-miR-2284ab	30.89	1.57	1.570E − 02
	chi-miR-3432-5p	211.51	1.28	1.570E − 02
	bta-miR-92b	3862.21	0.97	1.570E − 02
	chi-miR-1249	730.37	1.30	1.753E − 02
	chi-miR-1343	668.74	1.21	1.753E − 02
	new-miR-1306-5p	53.87	9.89	1.753E − 02
	oar-let-7b	46,112.69	1.14	1.753E − 02
	chi-miR-423-5p	22,808.97	1.48	1.864E − 02
	chi-miR-296-3p	954.25	1.12	2.036E − 02
	bta-miR-193a-5p	299.21	1.07	2.405E − 02
	bta-miR-744	611.97	1.09	2.405E − 02
	chi-let-7e-5p	4276.93	0.95	2.405E − 02
	chi-miR-483	27.04	1.51	3.988E − 02
	chi-miR-197-3p	2765.68	1.12	4.032E − 02
	chi-miR-328-3p	818.50	0.98	4.032E − 02
	oar-miR-432	139.69	1.24	4.521E − 02
	oar-miR-154b-5p	29.00	1.19	4.545E − 02
	oar-miR-16b	38,183.40	1.56	4.780E − 02
Down-regulated	chi-miR-148b-3p	2082.12	− 1.19	4.082E − 04
	oar-miR-29a	1881.46	− 1.92	1.279E − 02
	chi-miR-186-5p	19,718.93	− 1.00	1.570E − 02
	chi-miR-192-5p	6575.75	− 1.46	1.570E − 02
	oar-miR-27a	831.13	− 1.95	1.570E − 02
	oar-miR-30c	3695.69	− 0.89	1.570E − 02
	bta-miR-339a	1098.16	− 0.84	1.570E − 02
	chi-miR-181c-5p	674.55	− 1.64	1.570E − 02
	chi-miR-101-3p	447.85	− 1.51	1.753E − 02
	chi-miR-144-3p	34.17	− 4.15	1.753E − 02
	chi-miR-181c-3p	56.94	− 2.39	1.753E − 02
	chi-miR-338-3p	31.40	− 1.98	1.753E − 02
	chi-miR-660	830.22	− 1.10	1.753E − 02
	chi-miR-30e-3p	940.17	− 1.34	1.864E − 02
	oar-miR-99a	2175.87	− 1.39	2.086E − 02
	chi-miR-126-5p	1686.39	− 1.15	2.137E − 02
	chi-miR-126-3p	714.65	− 1.42	2.386E − 02
	oar-miR-148a	4231.97	− 1.37	2.405E − 02
	chi-miR-29a-3p	19.62	− 2.71	3.869E − 02
	oar-miR-30b	447.17	− 1.14	3.971E − 02
	chi-miR-335-3p	44.50	− 1.13	4.032E − 02
	oar-miR-143	757,185.06	− 1.15	4.299E − 02
	chi-miR-30e-5p	3764.20	− 0.94	4.397E − 02
	bta-miR-142-5p	1845.75	− 1.39	4.805E − 02
	chi-miR-199b-5p	31.33	− 1.87	4.805E − 02
	chi-miR-199c-5p	31.33	− 1.87	4.805E − 02
	chi-miR-27b-3p	51,268.06	− 0.77	4.999E − 02

**Table 2:** Differentially expressed miRNAs in the Adj vs. Control comparison. baseMean: average of the normalized DESeq2 count values.

Type	miRNA	baseMean	log2FoldChange	padj
Up-regulated	bta-miR-193a-5p	299.21	1.27	4.173E − 02
	chi-miR-1343	668.74	1.17	4.369E − 02
	oar-miR-485-5p	54.47	1.16	4.369E − 02
	bta-miR-92b	3862.21	0.86	4.546E − 02
	chi-miR-214-3p	3044.51	0.67	4.710E − 02
	chi-miR-3432-5p	211.51	1.08	4.896E − 02
Down-regulated	chi-miR-148b-3p	2082.12	− 1.02	8.400E − 03
	chi-miR-338-3p	31.40	− 2.10	4.173E − 02
	bta-miR-339a	1098.16	− 0.78	4.369E − 02
	chi-miR-186-5p	19,718.93	− 0.91	4.369E − 02
	chi-miR-223-3p	164.90	− 1.49	4.369E − 02
	chi-miR-30e-3p	940.17	− 1.37	4.369E − 02
	chi-miR-451-5p	10,575.83	− 2.18	4.369E − 02
	oar-miR-30c	3695.69	− 0.81	4.369E − 02
	chi-miR-126-5p	1686.39	− 1.12	4.710E − 02
	chi-miR-425-5p	724.73	− 0.69	4.710E − 02

### miRNA target prediction and analysis

**Figure 2:**
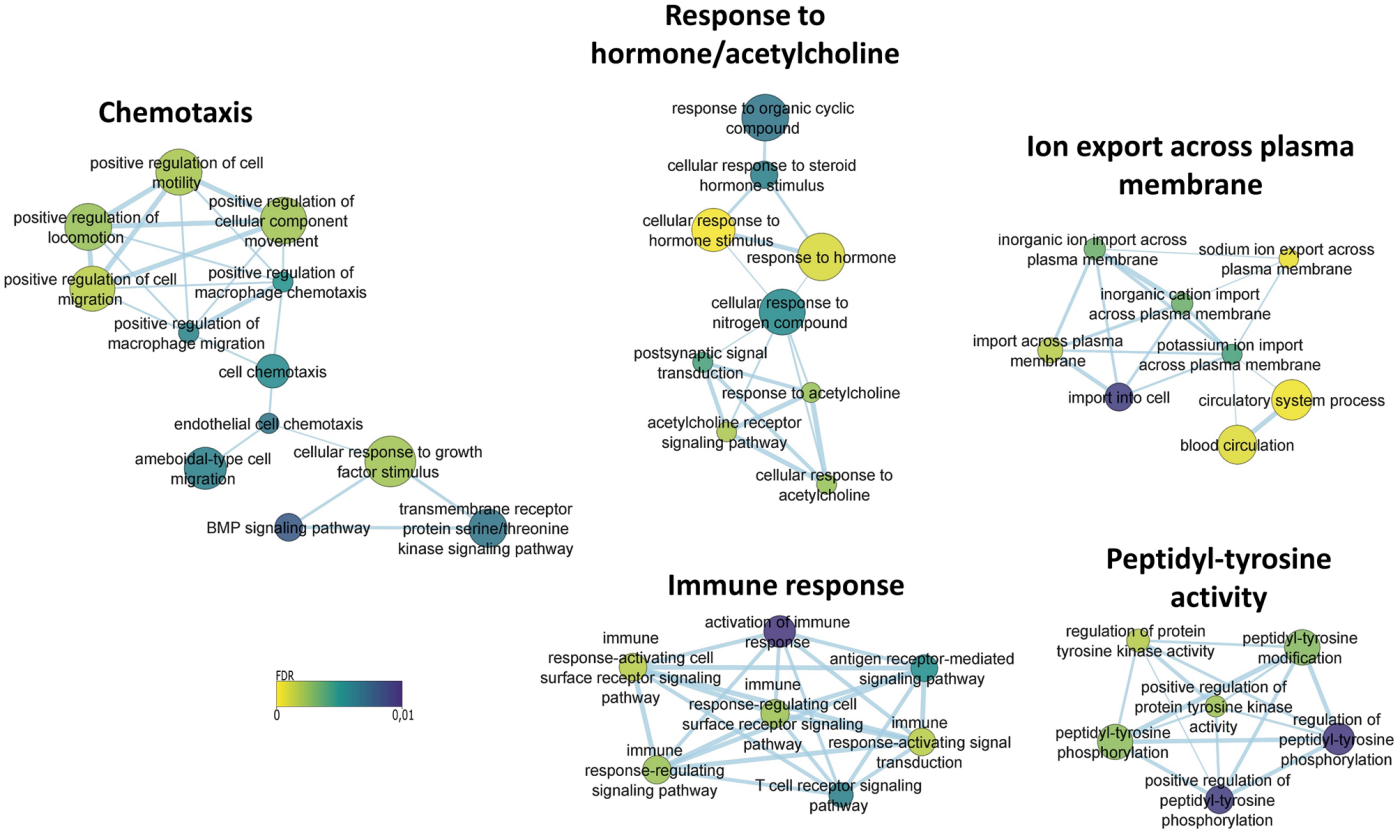
Enriched GO terms from the biological process category among the 75 most expressed miRNAs in spleen samples. Node size represents the number of targets in the term; edge size represents the number of targets that overlap between terms; node colour represents the significance level (adjusted p-value by the Benjamini–Hochberg method).

miRNA target prediction was performed with four different tools (miranda, pita, targetscan and tarpmir). The 3’ UTR sequences described in the Oar_rambouillet_v1.0 reference annotation file were used, which may be scarce compared with the annotation of 3’ UTR sequences in human and other organisms. 1637 targets were predicted for the 394 miRNAs in this study (Supplementary Fig. S5) and a functional enrichment analysis was performed with the predicted targets of the 75 most expressed miRNAs (Fig. 2). Among the significant GO terms, the most expressed miRNAs in spleen were related to positive regulation of chemotaxis with terms such as *positive regulation of macrophage chemotaxis* (GO: 0010759), *positive regulation of cell motility* (GO: 2000147) and *positive regulation of cellular component movement* (GO: 0051272). There were also several terms related to the immune response, such as *antigen receptor-mediated signaling pathway* (GO: 0050851), *immune response-activating signaling transduction* (GO: 0002757) and *T cell receptor signaling pathway* (GO: 0050852), among others.

The differentially expressed miRNAs were predicted to target multiple genes related to the immune system. For instance, cytokine receptors such as IFNGR2 (targeted by miR-193a-5p and miR-296-3p), IL1RAP (targeted by miR-432) and ENSOARG00020011968 (orthologous to the human TNFRSF10D and targeted by miR-339a); cytokines such as IL13 (targeted by let-7b, let-7e-5p and miR-328-3p) and CSF1 (targeted by miR-423-5p); serine/threonine-protein kinases such as RPS6KA5 (targeted by miR-744) and RIPK1 (targeted by miR-296-3p); and genes related to the NF-κB protein complex such as CASP8AP2 (targeted by miR-197-3p and miR-328-3p) and BCL3 (targeted by miR-296-3p) were among the predicted targets. A detailed list of the targets of the differentially expressed miRNAs can be seen in Table 3.

**Table 3:** Predicted targets of the differentially expressed miRNAs.

miRNA	Predicted target
bta-miR-193a-5p	POLR2H, CDK2, SYNDIG1, IFNGR2, TVP23B, ZZEF1, ARAP3, RARB, SLC13A2, ACAA1, PRSS27
bta-miR-339a	ENSOARG00020000740, PHC3, MX2, ILVBL, ITSN1, AGFG1, MBTPS1, PSMA6, SELENON, ENSOARG00020008854, PANK1, ENSOARG00020009052, ENSOARG00020011968, DAAM2, SIPA1L2, BDKRB2, DOCK2, PPP1R9A, VAT1, SYNRG, PRSS53, POLR3H, ATP6V1H, RGS20
bta-miR-744	KCTD17, ZNF699, ADGRE5, NCS1, NRBP1, WDR1, MYOD1, TAGLN, ARF5, KIF13B, NFIX, SPSB4, ALDH4A1, ARHGEF10L, SLC12A7, ZNF236, TCN2, RPS6KA5, SDC1, ENSOARG00020018455, BCAT2, GYS1, SEPTIN1, DRD2, ENSOARG00020021658, ATP6V0C
bta-miR-92b	TCF21
chi-let-7e-5p	IL13, IRS2, PGAM2, ADRB3, DTNA, UNC119, PRTG
chi-miR-101-3p	PANK3
chi-miR-1249	PSKH1
chi-miR-1343	ENSOARG00020001979, FBXO10, UBAC1, ENSOARG00020008683, RPS11, SORCS2, ABAT, PI4KB, MAU2, THAP3, FAM155B, ZSWIM8, TOP3A
chi-miR-148b-3p	BMT2, MED12L, ADAMTS1, PDE4D, FMR1
chi-miR-181c-3p	TBC1D20
chi-miR-197-3p	ANKRD54, CASP8AP2, PSMD11, PARG, ACVR1, STK38
chi-miR-199b-5p	REG4, RANBP2, KL, SULF1, TMEM163
chi-miR-199c-5p	REG4, RANBP2, KL, SULF1, TMEM163
chi-miR-27b-3p	ENSOARG00020009390, STK32A
chi-miR-296-3p	FSTL1, BCL3, RIPK1, IFNGR2, ZPBP, RUNDC3A, PRND, APIP, YPEL2, TOMM6, GABBR1, SH3PXD2B, MLN, ENSOARG00020018942, ADSS1, ENSOARG00020021320, DIPK2B, SMG6, NIPAL4
chi-miR-30e-3p	ENSOARG00020007190, SESTD1
chi-miR-30e-5p	ENSOARG00020021482
chi-miR-328-3p	IL13, POLDIP3, CASP8AP2, GLB1L, SLC6A13, PIGU, RAB15, ENSOARG00020016596, ARAP3, SRD5A1
chi-miR-335-3p	ATP6V1G1
chi-miR-338-3p	NIT2, PM20D1, ENSOARG00020014483, NRP1, ENSOARG00020023185
chi-miR-3432-5p	KCTD17, IRS2, FDXACB1, GALNT1
chi-miR-423-5p	KAT2B, PVALB, ENSOARG00020000307, KCTD17, S100A16, PROM2, ENSOARG00020001068, SSR3, LSMEM1, GLUL, CDK20, FAM234B, EIF2B1, CDK2AP1, RASSF3, CSF1, THBS3, HNF4A, CERS2, SLC38A7, ANPEP, TRPM2, SRM, ACHE, ST6GALNAC2, FXYD1, RAB15, CLEC16A, CNBP, LASP1, ENSOARG00020018455, TTLL5, HPCA, RHOBTB2, LPO, DDX49, FRMPD3, AGPS, ENSOARG00020024099, PHF8, CRKL
chi-miR-483	VAV3, ATP2B1, NUAK1, GALNT6, THRB, ENSOARG00020024411
chi-miR-660	FBXL5, NSD3
oar-let-7b	IL13, IRS2, KIN, UNC119
oar-miR-143	SECISBP2L, ATP10B, NFATC1, TRHR
oar-miR-148a	MED12L, FMR1, SGCB, NBR1
oar-miR-154b-5p	TBRG4, HFE, BMX, NOL10, TBC1D14, PCDHAC2, PLTP, ABHD11, PLEKHA1, WNK4
oar-miR-16b	CCNT2
oar-miR-27a	ENSOARG00020009390, PRIM2, STK32A
oar-miR-432	KIAA0825, TSHR, PDE1A, IL1RAP
oar-miR-485-5p	PTMS, VTA1, GOT2, MECR, PPP2R1B, THEMIS2, MRPS27, C18orf25, SFTPA1, DNAJA3
chi-miR-214-3p	HAO2, SCG5, KPNA3, TLE3, KIF1B, VAT1L, CAMK2G, AATF, CLCN7
chi-miR-425-5p	LIN28B

The intersection of four different tools (miranda, pita, targetscan and tarpmir) were used for miRNA target prediction.

### Predicted protein–protein interaction networks

**Figure 3:**
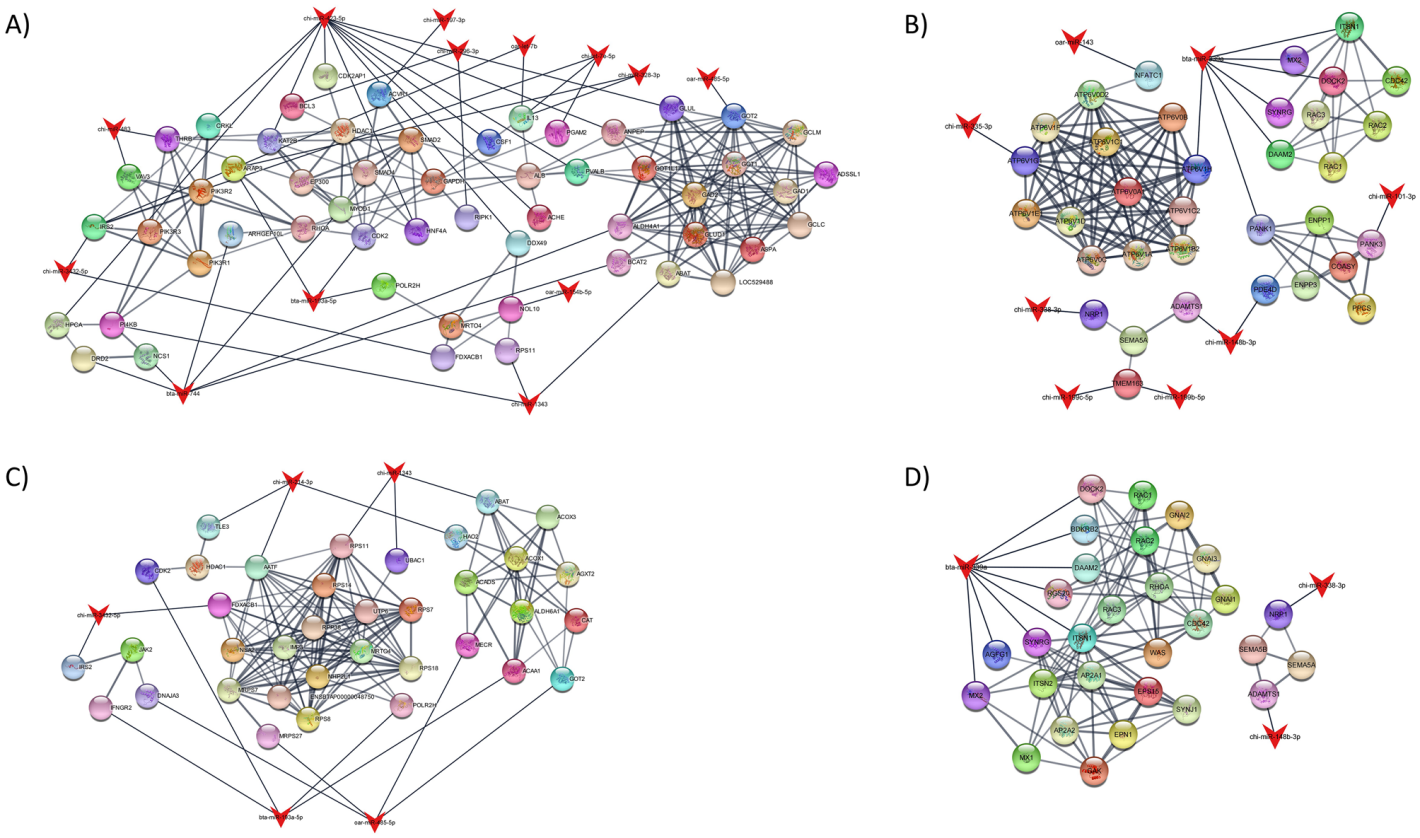
Protein–protein interaction networks. miRNAs are represented as red triangles in the network. (**A**) Network from the targets of the up-regulated miRNAs in the Vaccine vs. Control comparison; (**B**) Network from the targets of the down-regulated miRNAs in the Vaccine vs. Control comparison; (**C**) Network from the targets of the up-regulated miRNAs in the Adjuvant vs. Control comparison; (**D**) Network from the targets of the down-regulated miRNAs in the Adjuvant vs. Control comparison.

Protein–protein interaction (PPI) networks were constructed from the targets of the differentially expressed miRNAs, for each comparison and separating up-regulated and down-regulated miRNAs (Fig. 3A–D). A detailed list of the enriched terms in each network can be seen in Table S2A–D. There were multiple terms related to immunity in the network formed from the targets of the up-regulated miRNAs in the Vaccine and Control comparison. For instance, TNF signaling pathway (FDR = 1.75E − 5), FoxO signaling pathway (FDR = 2.33E − 5), Fc epsilon RI signaling pathway (FDR = 2.33E − 5), T-cell receptor signaling pathway (FDR = 9.27E − 5), and leukocyte transendothelial migration (FDR = 1.1E − 4). In contrast, there was no term related to immunity in the network from the targets of the up-regulated miRNAs in the Adjuvant and Control comparison. Regarding the network from the targets of the down-regulated miRNAs, there were multiple terms in common in both comparisons, such as Wnt signaling pathway, B cell receptor signaling pathway and natural killer cell mediated cytotoxicity in both comparisons. In Addition, oxidative phosphorylation (FDR = 3.9E − 18) and mTOR signaling pathway (FDR = 1.4E − 10) were enriched in the network from the targets of the down-regulated miRNAs in the Vaccine and Control comparison, while chemokine signaling pathway (FDR = 5.49E − 7) and leukocyte transendothelial migration (FDR = 8.96E − 7) were enriched in the Adjuvant and Control comparison.

### Validation by RT-qPCR

To validate the miRNA-seq data, 5 miRNAs (let-7b, miR-27a, miR-29a, miR-101-3p and miR-193a-5p) were also analyzed by RT-qPCR. Fold changes (FC) for these miRNAs expression as calculated by RT-qPCR and miRNA sequencing are shown in Supplementary Fig. S6. Although there was not statistically significant differential expression in the RT-qPCR analysis (t-test), the miRNA expression data from miRNA-seq and RT-qPCR showed concordance in the log2FC direction.

## Discussion

In this work, a total of 278 miRNAs were detected in ovine spleen, among them 75 previously annotated in sheep in miRBase, 194 homologous to other ruminant miRNAs, and 9 previously undetected novel miRNAs. The expression level distribution of the miRNAs was skewed, with the 10 most expressed miRNAs contributing up to 90% of the expression in each sample. The most expressed miRNA was miR-486, a miRNA reported to sustain the NF-κB pathway by suppression of multiple NF-κB-negative regulators^16^. This non-canonical miRNA is a marker of red blood cells^17^. Moreover, comparing the spleen with previously analysed tissues from the same group of animals^14,15^ the overlap among the different datasets was quite high with 110 miRNAs in common. The spleen showed the highest number of specific miRNAs (50% of the detected miRNAs in this tissue) and members of the let-7 family and miR-26a were highly expressed in all tissues in a consistent way^18^.

Among the 12 miRNAs dysregulated by both treatments, the downregulated miR-148b was the most significantly differentially expressed miRNA. This miRNA is involved in several functions in relation to immunity as a member of the miR-148/-152 family^19^. It has shown to be a negative regulator of cytokine production, antigen presentation and MHC II expression in mouse dendritic cells^20^. Its downregulation should indicate higher expression levels of the target gene *CAMK2A,* which, in turn, would activate the mentioned functions. Other dysregulated miRNAs in common such as miR-126-5p, miR-30e-3p and miR-186-5p have been related with the inflammatory signal^21–23^, pointing towards an active response to the vaccines and the adjuvant in spleen. There were few miRNAs differentially expressed after aluminium adjuvant-only treatment, and only four were exclusive to that treatment, even if we did not find any DE miRNA between both treatment groups. One of those miRNAs was the highly expressed miR-451, which is induced by influenza virus infection in murine splenic DCs and regulates DC cytokine production^24^ and response to influenza vaccine^6^.

Regarding the miRNAs dysregulated exclusively by the vaccine treatment, probably due to addition of antigen particles, we found that multiple miRNAs were previously reported to be related to macrophage polarization. For instance, miRNA let-7b-5p has been shown to regulate the polarization of macrophages to the M2 phenotype and regulate inflammatory cytokine expression through the SOCS1/STAT pathway^25^. Overexpression of miR-29a-3p has also been shown to enhance M2 subtype macrophage polarization via oral squamous cell carcinoma-derived exosomes and silencing of the miRNA has led to suppressed M2 polarization^26^, while its overexpression has been shown to supress Th1 cell differentiation via granulocytic myeloid-derived suppressor cell exosomes^27^. Moreover, the expression of miR-27a decreased with M1-associated cytokine stimulation and increased with M2-associated cytokine stimulation, pointing towards an involvement of the miRNA in the M1/M2 polarization^28,29^. Finally, miR-101-3p was shown to drive a proinflamatory phenotype in human macrophages by targeting the pseudokinase TRIB1^30^.

Multiple subsets of macrophages exist in the spleen^13^ and some may have important roles bridging the innate and adaptive immunity^31^. The macrophage activation states have been mainly studied in bone marrow macrophages, but spleen macrophages are also able to reprogram between the M1 and M2 activation states in mice^32^, even if differences between macrophage activation exist in livestock species^33^. The dysregulated miRNAs are candidates for further research since they might play key roles in the immune response induced by aluminium adjuvants^34^. A few cytokines, cytokine receptors and genes related to the NF-κB complex were possibly targeted by the differentially expressed miRNAs.

As for the protein–protein interaction is concerned, there are some pathways common in both vaccinated-control and adjuvanted-control comparisons. The B cell receptor signalling pathway for example arises in both comparisons. Part of the spleen allows generation of antigen-specific immune responses that protect the body against blood-borne bacterial, viral and fungal infections. Additionally, the spleen is a site where immune responses that are deleterious to the host can be regulated. As a major cell type in spleen, B cells play a crucial role in regulating immune response. Chen et al.^35^ reported that the absence of B cells almost fully inhibited the recruitment of dendritic cells, neutrophils, and macrophages to the peritoneal cavity during pristine-induced chronic inflammation^35^. Later, the same group observed a similar regulatory pattern in the spleen^36^.

On the other hand, other pathways were differently regulated by miRNAs in the comparisons analysed. In the protein–protein interaction networks, leukocyte TEM was found enriched in the network from the targets of the up-regulated miRNAs in vaccinated animals, while it was also enriched in the adjuvanted animals but in the network from the targets of the down-regulated miRNAs. Inflammation is tightly regulated and is associated with the transient crossing of leukocytes through the blood vessel wall, a process called transendothelial migration (TEM)^37^. The scope and speed of the innate immune response is primarily dictated by TEM^38^. It is interesting that TEM had been activated in the mobilization of leucocytes from spleen to other tissues in the adjuvanted animals, but not in the vaccinated animals, so we can hypothesize that different cellular migrations occur depending on the inoculated molecules. More information is needed to disentangle this mobilization mechanism.

To sum up, the expression changes in the spleen miRNAome of sheep experimentally treated with vaccines have been analysed for the first time. Animals were treated either by commercial vaccines, aluminium hydroxide diluted in PBS or PBS alone. Several miRNAs were found differentially expressed. In the same fashion as in PBMCs^14^, both aluminium hydroxide alone and commercial vaccines altered the miRNA expression, but the impact of aluminium hydroxide was smaller in the spleen. Histopathological parameters remained within normal values in splenic tissue and repetitive administration of both treatments did not cause major adverse reactions after the experiment^39^. Therefore, the transcriptomic changes observed in these and previous results reflect a normal immune response to vaccine adjuvants.

## Material and methods

### Ethics statement

All experimental procedures were approved and licensed by the Ethical Committee of the University of Zaragoza (ref: PI15/14). Requirements of the Spanish Policy for Animal Protection (RED53/2013) and the European Union Directive 2010/63 on protection of experimental animals were always fulfilled.

All methods are reported in accordance with ARRIVE guidelines (https://arriveguidelines.org) for the reporting of animal experiments.

### Animals

Animals analysed in this work were included in a long-term experiment. Briefly, three-month-old Rasa Aragonesa purebred lambs were selected from a single pedigree flock, with the condition they had not previously undergone any kind of vaccination. Twenty-one animals were established at the experimental farm of the University of Zaragoza and were always kept indoors with controlled conditions of housing, management and diet. After a two-month acclimatization period, the animals were randomly distributed in three different treatment groups, each consisting of seven animals. In one of the groups, which was denominated the vaccine group (Vac), animals were administered aluminium-based subcutaneous commercial vaccines. In another group, denominated adjuvant group (Adj), animals were treated with equivalent doses of aluminium hydroxide (Alhydrogel, CZ Veterinaria, Spain) diluted in phosphate-buffered saline (PBS). Finally, PBS was administered to the control group. The experiment lasted 475 days, from February 2015 to June 2016, and nine different vaccines were administered covering 19 inoculations across 16 different inoculation dates. A detailed list of the vaccination schedule can be seen as supplementary material in a previous publication^14^.

### Tissue collection and RNA extraction

Samples from spleen were aseptically taken from each animal at necropsy and tissue sections were preserved in RNAlater solution (Ambion, Austin, TX, USA) at − 80 °C. Samples from spleen were always fragments of esplenic parenquima, including both red and white pulp. The experimental procedure to obtain RNA was similar to the one previously performed in the analysis of parietal lobe cortex^15^. Total RNA was isolated from spleen tissue using TRIzol Reagent (Invitrogen, Carlsbad, CA, USA) and PureLink RNA Mini Kit (Invitrogen). 60 mg tissue samples were homogenized in 1 ml of TRIzol Reagent using Precellys24 homogenizer (Bertin Technologies, Montigny-le-Bretonneux, France) combined with 1.4- and 2.8-mm ceramic beads mix lysing tubes (Bertin Technologies). RNA isolation was performed following manufacturer instructions and RNA was suspended in RNase free water and stored at − 80 °C. RNA quantity and purity was assessed with NanoDrop 1000 Spectrophotometer (Thermo Scientific Inc, Bremen, Germany). RNA integrity was assessed on an Agilent 2100 Bioanalyzer with Agilent RNA 6000 Nano chips (Agilent Technologies, Santa Clara, CA, USA), which estimates the 28S/18S (ribosomic RNAs) ratio and the RNA integrity number (RIN value). The samples presented a RIN value > 7.6 and a 260/280 ratio > 2.02.

### miRNA sequencing

Twelve spleen samples were sequenced, four from each group. The TruSeq Small RNA library prep kit (Illumina) was used for miRNA sequencing (miRNA-seq). The libraries were sequenced on a HISeq2500 with a mean sequencing depth of 14.9 million reads (50 bp single-end reads) at CRG (Centro de Regulación Genómica, Barcelona, Spain).

### Differential expression analysis

Adapter trimming and low-quality sequence trimming was performed with Trimmomatic [v0.39]^40^. Reads with an average Phred quality score within a sliding window of five nucleotides below 20 or with a length sorter than 16 nucleotides were removed. Then, rRNA reads were removed with bbduk from bbtools [v39.90] (https://sourceforge.net/projects/bbmap/) and with the rRNA SILVA database (release 138.1). The remaining reads were aligned to the *Ovis aries* reference genome Oar_rambouillet_v1.0, allowing a maximum of 20 multiple mappings per read, and miRNAs were characterized searching against the miRBase database with the srnabench module from srnatoolbox^41^. In addition to sheep miRNAs from miRBase, sequences were screened for caprine and bovine miRNAs, with a priority of search ovine > caprine > bovine. The reads that were not assigned to any ruminant miRNA were used for novel miRNA characterization with srnabench following author recommendations. Once expression estimates were achieved, a differential miRNA expression analysis was performed with DESeq2 [v1.32.0]^42^. Only miRNAs with a minimum expression of 1 count per million (cpm) in at least four samples were taken as truly expressed. Those miRNAs with an adjusted p-value (with the Benjamini–Hochberg method) threshold of < 0.05 and a fold change > 1.5 or < 0.667 were selected as differentially expressed.

### Target prediction

Four different algorithms were used for the miRNA target prediction, targetscan^1^, tarpmir^43^, miranda^44^ and pita^45^, with the latter two implemented in the mrnaconstarget module from srnatoolbox. The following filtering criteria were applied to select trustworthy target genes: (i) in miranda, a pairing score > 155 and an energy score < − 20; (ii) in pita, an energy score < − 20; (iii) in targetscan, a weighted contex ++ score percentile > 90; (iv) in tarpmir, a binding probability > 0.5. The intersection of the four target prediction algorithms was taken as reliable target candidates to deal with the inherent high false positive rate of these programs.

The predicted targets were further analysed by a gene enrichment analysis against the GO terms by g:Profiler^46^. The targets of the 75 most expressed miRNAs were used in the enrichment to study the candidate functions of the most expressed miRNAs in spleen. g:profiler is a tool that computes p-values for enriched terms using a Fisher’s exact test and the Benjamini–Hochberg method was selected for multiple test correction. Those terms with an adjusted p-value less than 0.01 were selected. Moreover, the terms were further analysed within Cytoscape using Enrichmentmap and Autoannotate plugins^47^. The Enrichmentmap plugin generates a network in which terms are seen as nodes and are connected between each other if they share multiple genes. For visualization purposes, terms composed of more than 400 genes or less than 5 genes were removed from the analysis due to their limited interpretative value. The Autoannotate plugin finds clusters within the network and visually annotates them with labels based on word frequencies. Those labels were later manually curated. Clusters with less than 4 interconnected nodes were removed for visualization purposes.

### Protein–protein interaction network

A protein–protein interaction network analysis was performed with the targets of the differentially expressed miRNAs. The stringApp [v1.7.1]^48^ plugin from Cytoscape [v3.9.1] was used for the network analysis. *Bos taurus* was used as the reference organism and only protein–protein interactions with a high confidence score (greater or equal to 0.7) were selected. Two interacting proteins in a PPI network may share a function or be involved in similar diseases. Thus, the maximum additional interactor, which determines the maximum number of additional interactions that its added to the targets of interest, was set to 20. This allows to construct an expanded network in which miRNA targets and their most common interactions are added. Clusters with 3 or less proteins were removed from the resulting network. Gene enrichment analyses were performed with the networks from the upregulated and downregulated miRNA targets for each comparison. Those terms with an FDR less than 0.05 were selected as significant.

### RT-qPCR

To validate changes identified by the miRNA sequencing, the expression levels of 5 miRNAs (let-7b, miR-27a, miR-29a, miR-101-3p and miR-193a-5p) were verified by RT-qPCR at the SGIKER platform of the UPV/EHU. The expression of miR-199 and miR-125b were used as internal standards. These miRNAs were selected due to the expression stability shown in our sequenced samples. Primers were designed using the Qiagen platform (see Table S3 for primer sequences). Three samples per group, independent from the sequenced ones, were used for RT-qPCR validation. The RNA was treated with the TURBO DNA-free kit from Invitrogen following manufacturer protocol. The resulting RNA was quantified in the NanoDrop 1000 spectrophotometer. The detection of the miRNAs was performed in the miRCURY LNA miRNA SYBR Green PCR system from QIAGEN following manufacturer instructions and amplicon detection was performed in SYBR Green fluorochrome. The RT-qPCR was performed in the CFX384 system from BioRad. Changes in miRNA relative quantification (RQ) were determined by the ∆(∆Ct) method. Comparison between vaccine and control groups was performed with a t-test upon data meeting normal distribution and homogeneity of variance.

## Supplementary Information

Supplementary Information.

Supplementary Table S1.

Supplementary Table S2.

Supplementary Table S3.

## Data Availability

miRNA sequencing data have been deposited in the NCBI Gene Expression Omnibus (GEO) database with experiment accession number GSE180596.
