# Supplementary material for: Identification and characterization of miRNAs in spleens of sheep subjected to repetitive vaccination
Source: Sci Rep. 2023 Apr 17;13:6239. doi: 10.1038/s41598-023-32603-7 (PMC10107569; doi:10.1038/s41598-023-32603-7)

Supplementary Material

Identification and characterization of miRNAs in spleens of sheep subjected to repetitive vaccination

Endika Varela-Martínez[1], Martin Bilbao-Arribas[1], Naiara Abendaño[1*], Javier Asín[2], Marta Pérez[2], Damian de Andrés[3], Lluis Luján[2] and Begoña M. Jugo[1]

[1]Department of Genetics, Physical Anthropology and Animal Physiology, Faculty of Science and Technology, University of the Basque Country (UPV/EHU), Leioa, Spain

[2]Department of Animal Pathology, Veterinary Faculty, University of Zaragoza, Zaragoza, Spain

[3]Institute of Agrobiotechnology (CSIC-UPNA-Government of Navarra), Mutilva, Spain

*** Correspondence:**
Begoña M. Jugo
e-mail: begonamarina.jugo@ehu.eus

1 Supplementary Figures and Tables

Table S1: Summary statistics from the alignment to the reference genome (Oar_rambouillet_v1.0) and from the miRNA characterization.

Table S2A-D: Enriched terms in the protein-protein interaction networks. A) Network from the targets of the up-regulated miRNAs in the Vaccine vs. Control comparison; B) Network from the targets of the down-regulated miRNAs in the Vaccine vs. Control comparison; C) Network from the targets of the up-regulated miRNAs in the Adjuvant vs. Control comparison; D) Network from the targets of the down-regulated miRNAs in the Adjuvant vs. Control comparison.

Table S3: List of the selected miRNAs and the corresponding primer sequences for the validation of the miRNA-seq.

Figure S1: The 10 most highly expressed miRNAs in each sample in peripheral blood mononuclear cells (PBMCs). In the x-axis the samples and in the y-axis the percentage of the total reads for each miRNA. The bars are coloured by miRNAs.

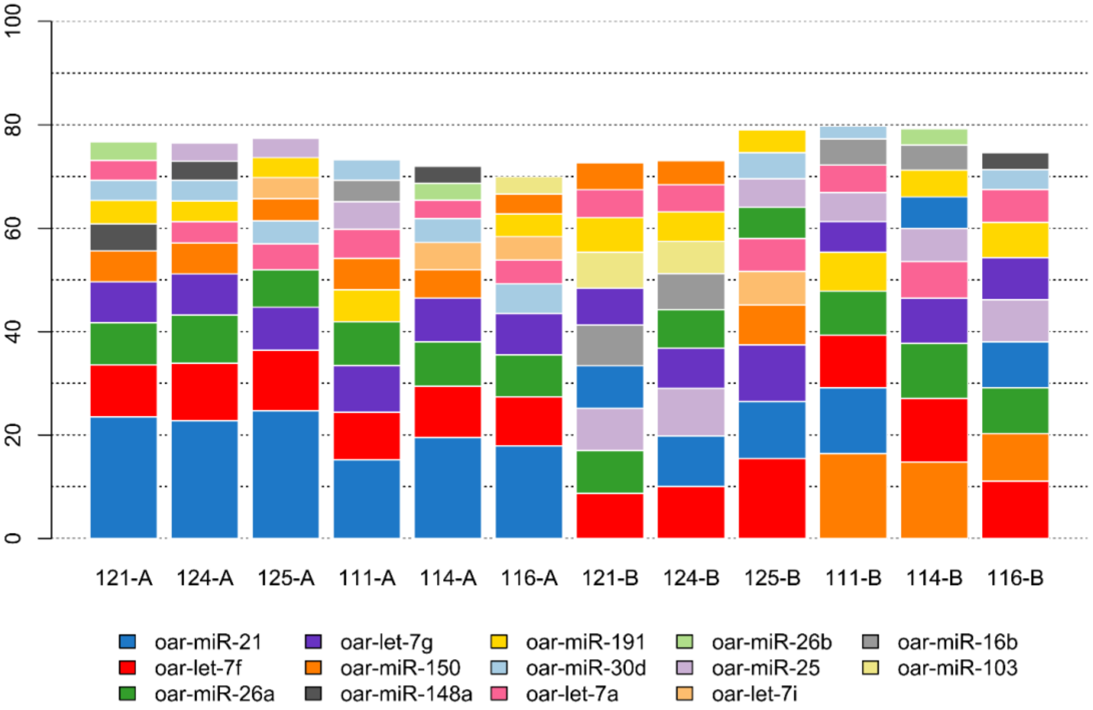

Figure S2: The 10 most highly expressed miRNAs in each sample in parietal lobe cortex. In the x-axis the samples and in the y-axis the percentage of the total reads for each miRNA. The bars are coloured by miRNAs.

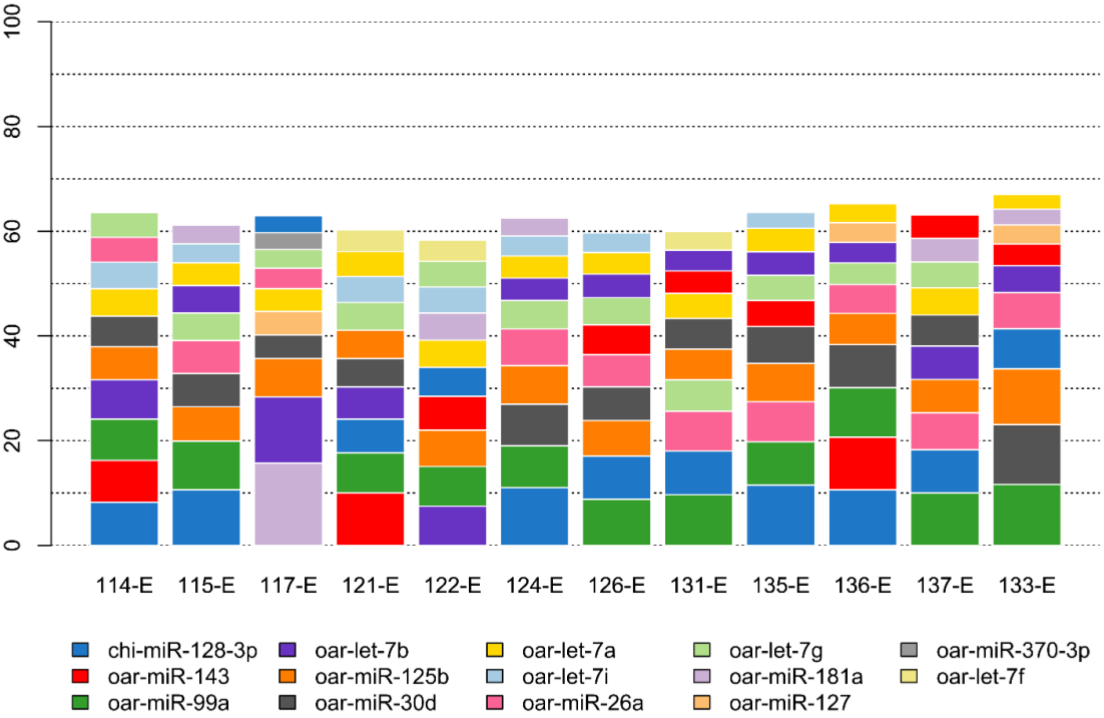

Figure S3: Venn diagram showing the intersection of the miRNAs detected in three different tissues (spleen, peripheral blood mononuclear cells and parietal lobe cortex) in the same group animals. The samples send for sequencing do not necessarily match between tissues.

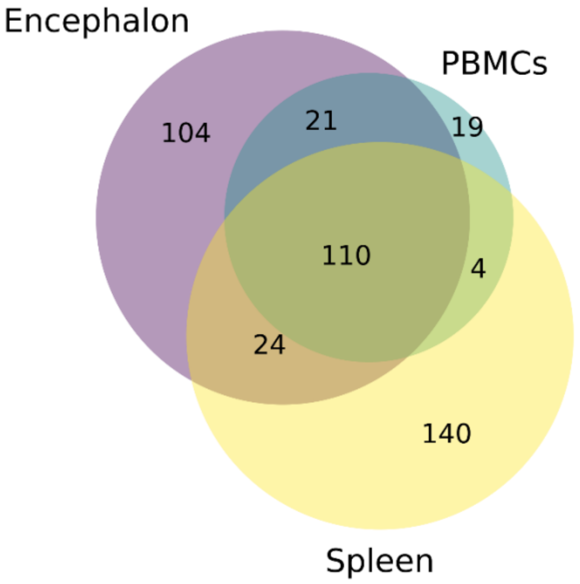

Figure S4: Principal Component Analysis (PCA) of the miRNA-seq data from animals treated with commercial vaccines (Vaccine group), with the adjuvant diluted in PBS (Adjuvant group) and with PBS (Control group).

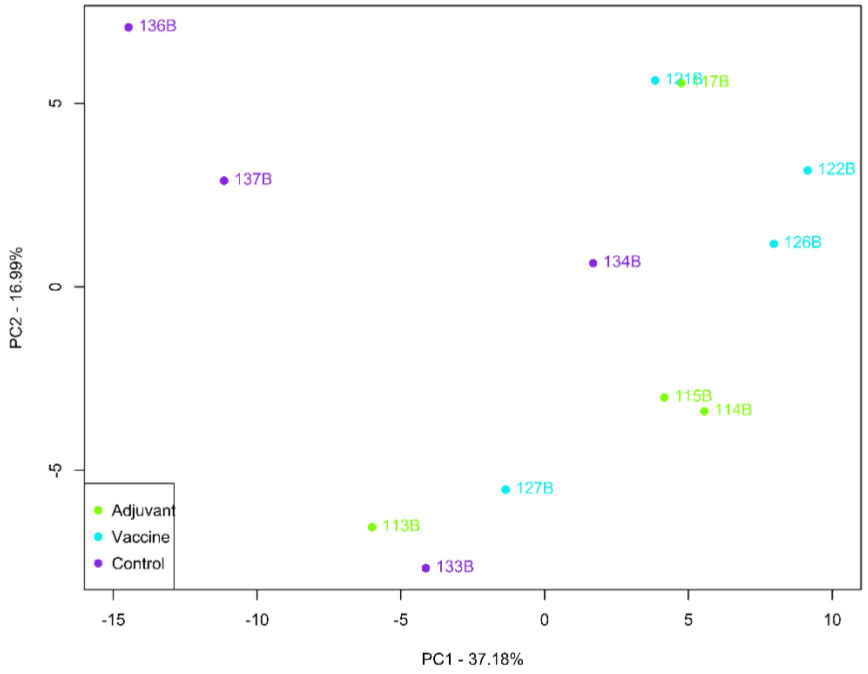

Figure S5: Venn diagram showing the intersections of the four miRNA target prediction algorithms (miranda, pita, targetscan and tarpmir).

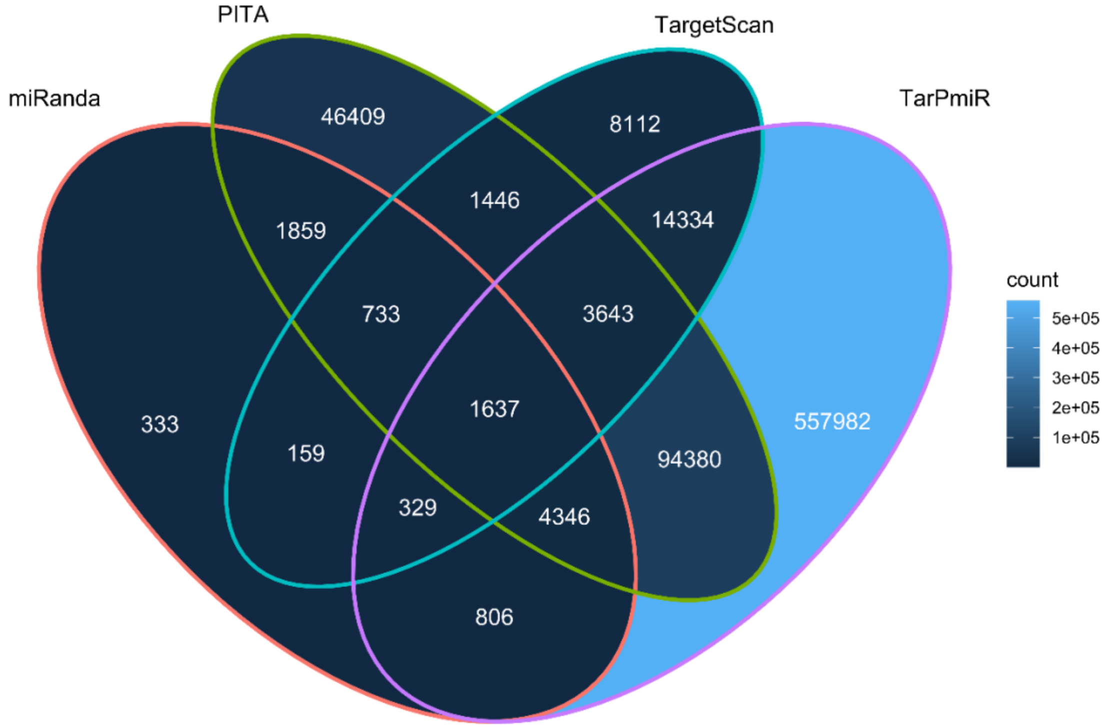

Figure S6: log2 Fold Changes (log_2FC) in miRNA expression as calculated by RT-qPCR and miRNA sequencing. Error bars correspond to the Standard Error of the Mean (SEM).

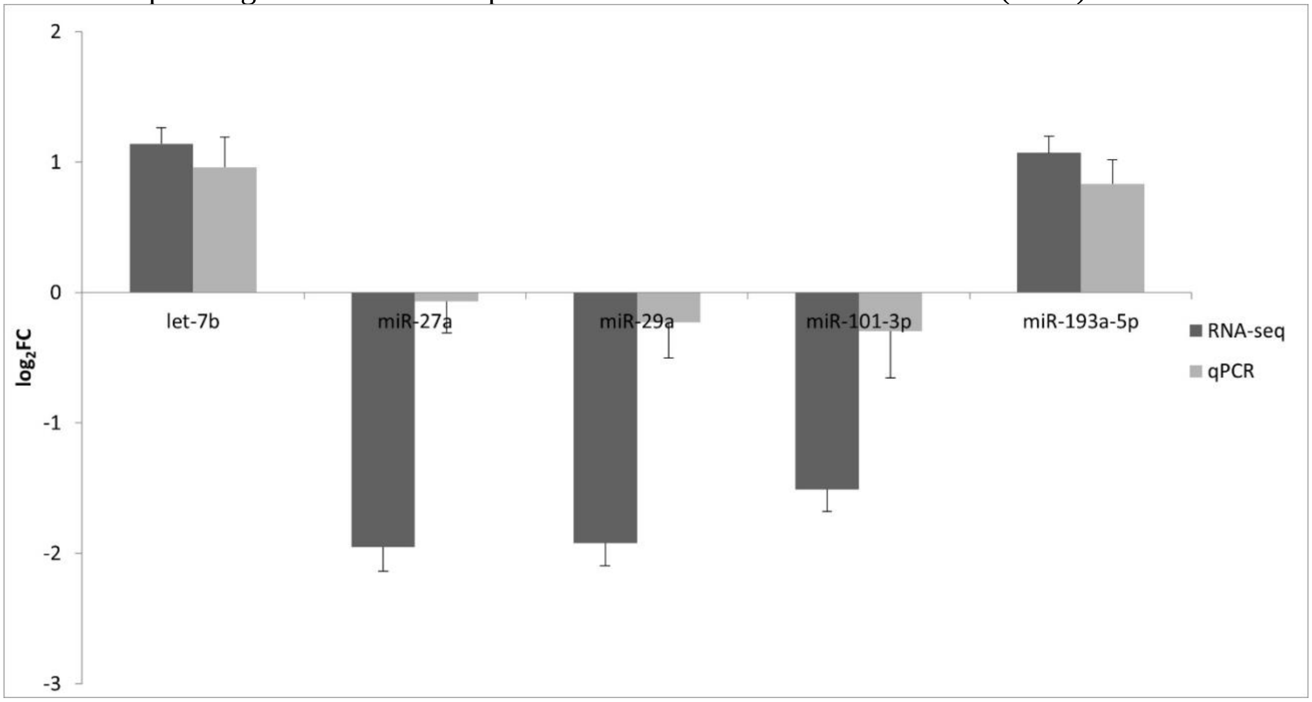

Supplement: Supplementary file 1 — Supplementary Information. [file 41598_2023_32603_MOESM1_ESM.pdf]
